# Man versus Machine: Software Training for Surgeons—An Objective Evaluation of Human and Computer-Based Training Tools for Cataract Surgical Performance

**DOI:** 10.1155/2016/3548039

**Published:** 2016-10-27

**Authors:** Nizar Din, Phillip Smith, Krisztina Emeriewen, Anant Sharma, Simon Jones, James Wawrzynski, Hongying Tang, Paul Sullivan, Silvestro Caputo, George M. Saleh

**Affiliations:** ^1^Moorfields Eye Hospital, 162 City Road, London EC1V 2PD, UK; ^2^Department of Computer Science, University Of Surrey, Guildford, UK; ^3^Moorfields Eye Hospital, Bedford, UK; ^4^NYU Langone Medical Center, New York, USA; ^5^Royal Free Hospital, London, UK; ^6^NIHR Biomedical Research Centre at Moorfields Eye Hospital and the UCL Institute of Ophthalmology, London, UK

## Abstract

This study aimed to address two queries: firstly, the relationship between two cataract surgical feedback tools for training, one human and one software based, and, secondly, evaluating microscope control during phacoemulsification using the software. Videos of surgeons with varying experience were enrolled and independently scored with the validated PhacoTrack motion capture software and the Objective Structured Assessment of Cataract Surgical Skill (OSACCS) human scoring tool. Microscope centration and path length travelled were also evaluated with the PhacoTrack software. Twenty-two videos correlated PhacoTrack motion capture with OSACCS. The PhacoTrack path length, number of movements, and total procedure time were found to have high levels of Spearman's rank correlation of −0.6792619 (*p* = 0.001), −0.6652021 (*p* = 0.002), and −0.771529 (*p* = 0001), respectively, with OSACCS. Sixty-two videos evaluated microscope camera control. Novice surgeons had their camera off the pupil centre at a far greater mean distance (SD) of 6.9 (3.3) mm, compared with experts of 3.6 (1.6) mm (*p* ≪ 0.05). The expert surgeons maintained good microscope camera control and limited total pupil path length travelled 2512 (1031) mm compared with novices of 4049 (2709) mm (*p* ≪ 0.05). Good agreement between human and machine quantified measurements of surgical skill exists. Our results demonstrate that surrogate markers for camera control are predictors of surgical skills.

## 1. Introduction

In recent years objective feedback for trainees in surgical procedures has become increasingly important for the training of new surgeons, not only to overcome the learning curve at an earlier stage, but also due to the environment of reduced training opportunities and stringent clinical commitments. Two broad systems have emerged, one is human and the other is machine based, but to date no study has compared the two on the same dataset.

One group of systems, termed the objective structured tools, are based on trainer led rating with tools which have defined scores. The Objective Structured Assessment of Cataract Surgical Skill (OSACSS) is one such tool and has been shown to statistically significantly discriminate between junior and senior surgeons [1]. The OSACSS has also been used as the platform for the construction of the International Council of Ophthalmology's Ophthalmology Surgical Competency Assessment Rubrics (ICO-OSCAR) scheme that is currently being applied worldwide and available to download for free online in a multitude of different languages [2]. The OSACSS was used as opposed to the ICO-OSCAR, as the original OSACSS study [1] included construct validation (the differentiation based on experience), which is directly related to the objectives of this study for comparing OSACSS with PhacoTracking and the OSACSS was designed as a quantitative rating tool. Conversely, ICO-OSCAR was designed as a formative feedback tool, designed to provide feedback and progress whilst learning and hence was less appropriate for this study.

The other group of systems used was motion analysis, allowing movements of a surgeon's hands, instrument, or camera view to be evaluated with metrics such as path length, time, and number of movements being derived. These machine based tools are purely quantitative and have also been shown to statistically significantly discriminate between different surgical skill levels [3, 4]. The PhacoTrack software is one example where computer vision tracking has been applied to phacoemulsification surgery and successfully differentiated between junior and senior surgeons [5]. This methodology has been applied to other ophthalmic procedures including endoscopic dacryocystorhinostomy surgery [6]. Motion tracking also underpins high fidelity simulators such as the EyeSi simulator (VRmagic Holding AG, Mannheim, Germany), which are growing in importance and relevance in eye surgery [7–10].

Key to both of these measurement tools is the concept of validity. By definition, validity provides an overall evaluative judgment of the degree to which empirical evidence and theoretical rationales support the* adequacy* and* appropriateness* of* interpretations* and* actions* based on test scores or other modes of assessment [11, 12]. The popularised Messick validity tool encompasses five different aspects emphasizing content, substantive, structural, external, and consequential aspects of construct validity. In effect, these* five* aspects function as general validity criteria or standards for all educational and psychological measurement. In this study, we examine instrument motion metrics relationships to other variables in one group and internal structure is investigated in a second group by investigating a subset of motion metrics regarding microscope control. PhacoTrack and OSACSS demonstrate validity for measuring surgical skill to varying degrees. Identifying differences in validity for each method will enable us to understand how such methods could be combined to provide more valid means of measuring surgical skill.

Objective structured systems are based upon the human experts' structured evaluation; in contrast measurements of skill through motion analysis utilise machine feedback. Whilst both have been independently validated these two distinct tools have not been previously applied to the same set of data; thus there is a poor understanding of their interrelationship. This study aimed to explore two related aspects. Firstly, to assess how both human and machine tools relate to each other in cataract surgery, this would help establish content and substantive validity and ascertain how well the two systems work together. Thereafter, the aim was to select a component of training that had not previously been specifically tested but was an explicit component of the human OSACSS tool that could be clearly tested by the PhacoTrack. Microscope control, which is an explicit stem of the OSACSS, was chosen. The applicability of microscope motion tracking in live cataract surgery between junior and senior surgeons, using the PhacoTracking tool (5), was then evaluated. The hypothesis was that those surgeons, who have a low path length and reduced number of movements as dictated by the PhacoTrack software, would have a higher OSACCS score. Equally, the hypothesis was that senior surgeons would keep the microscope in the central field of view with minimal movement compared with junior surgeons, as recorded by the PhacoTracking software.

## 2. Methods

Full IRB and ethics approval were awarded for this study.

### 2.1. Subjects

Videos from surgeons with varying experience were used in this study. A junior surgeon was defined as an operator with <200 completed cases whilst a senior surgeon was one with >1000 completed cases.

### 2.2. Videos

These were captured through the microscope-viewing platform with standard recording equipment, which captured the surgeons' operative perspective. All cataract cases were deemed suitable for junior surgeons to allow for a fair comparison.

The following inclusion and exclusion criteria were applied to all patients who had given informed consent, whilst undergoing routine phacoemulsification cataract surgery.

*Inclusion Criteria. *Inclusion criteria for both groups of systems included adult patients who had given informed consent, prior to undergoing routine phacoemulsification cataract surgery. Surgeons operated only on straightforward cases, and the inclusion criteria for patients were as follows: pupils fully dilating; mild to moderate cataract (1+ nuclear sclerosis or cortical lens opacity only); ability to fully lie flat and still for the duration of surgery; and no ocular comorbidity (e.g., glaucoma or pseudoexfoliation) [4].

*Exclusion Criteria.* Exclusion criteria included a patient unable to give informed consent or not wishing to participate; nonroutine cataract (e.g., secondary to previous trauma or previous intraocular surgery); concurrent pathology that would exclude a clear view (e.g., corneal pathology); and complex cases not suitable for the less experienced surgical grade (e.g., very small pupil, mature cataracts, and patients with pseudoexfoliation) [4].

*Group 1.* In this group the motion tracking software was compared with OSACSS on the same set of surgical videos.

**Figure 1:**
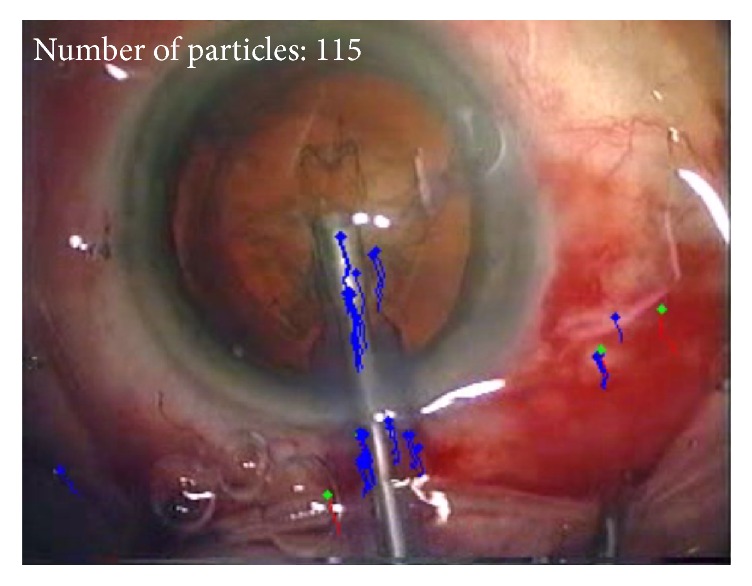
Screenshot of the PhacoTracking tool tracking the phacoemulsification probe.

*(1) Motion Analysis.* Software analysis using computer vision tracking methodology, which has been previously described in full, was employed [5]. A combination of SURF point detection [6] and Kanade-Lucas-Tomasi tracking [7] was applied to measure the motion of instruments used throughout the procedure in a fully automatic manner. The system analyses the full video of the surgery one frame at a time and measures the movement of each instrument within the field of view between frames. These measurements are used to calculate the instrument path length, the number of movements, and the total time accrued during each operation [5] (Figure 1). 

**Figure 2:**
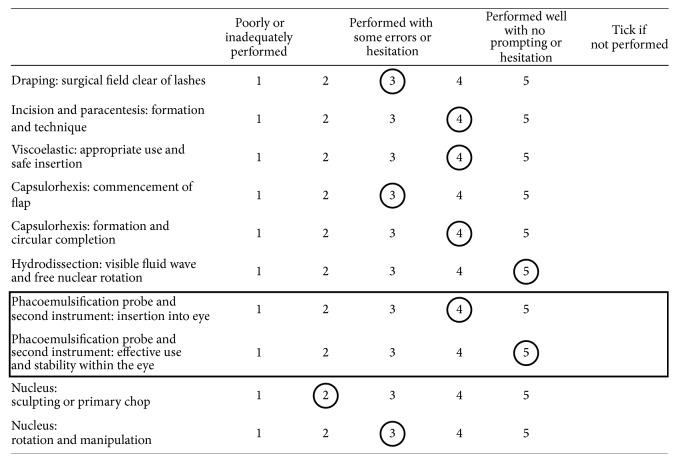
OSACCS scoring tool independently evaluating phacoemulsification.

*(2) OSACSS Scoring.* The same videos were anonymised, randomised, and then passed on to an independent expert (PS) who graded the skill level according to the objective structured assessment of cataract surgical skill (OSACSS) [1]. This tool consists of both global and phacoemulsification task-specific elements and is rated on a 5-point Likert scale totalling 100 potential points for the whole procedure (Figure 2) [1].

*Group 2*

**Figure 3:**
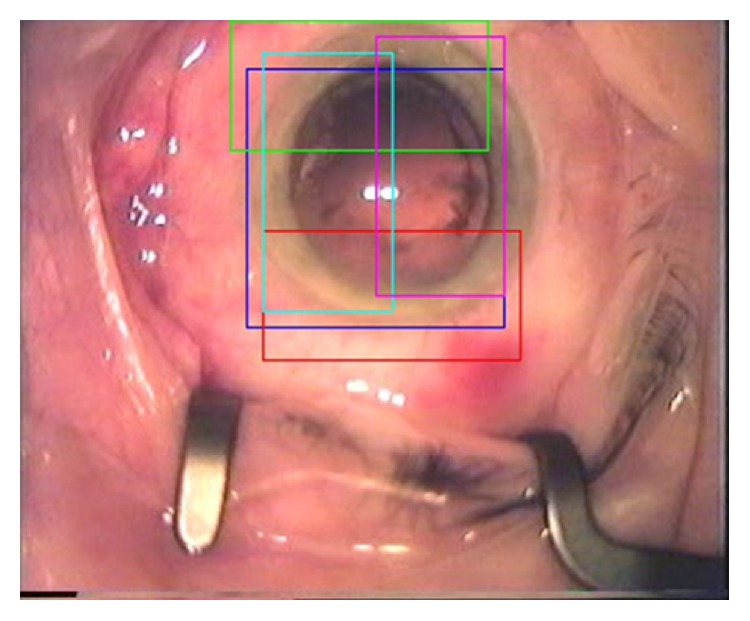
Snapshot of the iris tracking algorithm components.

*(1) Microscope Tracking.* In the second group of subjects, a computer vision algorithm that tracks the location of the iris was applied [7, 13]. A set of “histogram of orientated gradients” (HoG) detectors was applied to locate the pupil position [14]. Five different regions of the eye were detected in order to add robustness to the system (see Figure 3). These included superior, inferior, nasal, and temporal regions of the pupil, and an average of these values was computed to provide an estimate of the centre of the eye. From this tracking result, the distance from the centre of the pupil to the centre of the frame was then calculated for each frame along with the path length of the operative camera during the procedure. Similar analysis was used to calculate the total path length of the camera during the surgical video. The surgeons were split into novices (<200 cases) and experts (>1000 cases) with the camera tracking tool applied to each video.

*Statistical Analysis.* Mean, standard deviation (SD), and 95% confidence intervals were computed based on the recorded scores from each task. Spearman rank correlation was used to establish the relationship between OSACSS and PhacoTrack (for path length and movement).

Bland-Altman analysis comparing levels of agreement between the OSACCS and PhacoTrack ±1.96 standard deviation was undertaken. As the scale of both path length and number of movements is different than OSACCS, a linear model was found between the two measurements. The Bland-Altman plots use fitted OSACCS values for both path length and number of movements. The Bland-Altman provides a graphical representation of the levels of agreement/disagreement between the PhacoTrack and OSACSS within the defined margins. If there is little or no agreement then clinically they are not overlapping enough to make measurements comparable and usefully applicable to the same dataset. Conversely, if they agree 100% then there is no point in having two systems as they will be doing much the same thing.

A Mann–Whitney *U* test with significance at *p* < 0.05 was undertaken to test for a statistically significant difference in the total path length and mean decentration of the microscope between junior and senior surgeons. Python 2.7 and Scipy 1.9 statistical software were employed.

## 3. Results 

*Group 1.* In the first group of systems, comparing PhacoTracking instruments with OSACSS, 22 videos from 22 surgeons (11 novices, 11 experts) were enrolled and analysed in the study.

In all cases, there was a strong negative correlation meaning a higher OSACSS corresponded with fewer instrument movements, a reduced path length, and shorter operative time. A strong correlation between the natural logarithm of the number of instrument movements (as measured by PhacoTrack motion analysis) and the total OSACSS score was observed with Spearman's rank correlation coefficient giving a result of −0.6652021 (*p* = 0.002). This suggests that the number of movements was inversely proportional to the surgeon's OSACSS score. A correlation between the path length of the instruments travelled as measured by PhacoTrack motion analysis and the total OSACSS score was observed with Spearman's rank correlation coefficient giving a result of −0.6792619 (*p* = 0.001). This strongly suggests that the path length was inversely proportional to the surgeon's OSACSS score. The time taken for the procedure was inversely correlated to the score as measured by the OSACCS with Spearman's rank correlation of −0.771529 (*p* = 0.001). This strongly suggests that the time was inversely proportional to the surgeon's OSACSS score.

**Figure 4:**
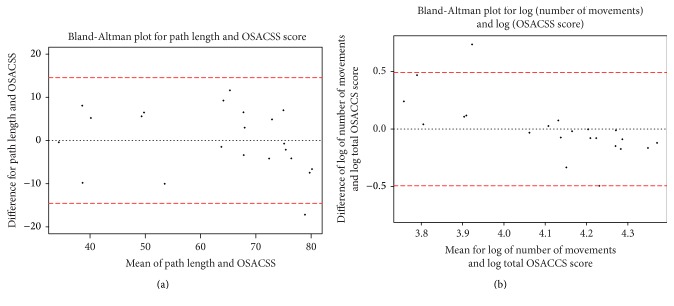
(a) Bland-Altman plot of OSACSS score to PhacoTrack path length (fitted by linear model to OSACSS score). Red lines represent the limits of agreement from −1.96s to +1.96s and dotted line is the mean difference. (b) Bland-Altman plot of Log OSACSS score to log PhacoTrack number of movements (fitted by linear model to OSACSS score). Red lines represent the limits of agreement from −1.96s to +1.96s and dotted line is the mean difference.

Figure 4 shows a Bland-Altman plot comparing the level of agreement for the OSACCS and PhacoTrack scores. The Bland-Altman plot describes the level of agreement between two quantitative measurements by constructing limits of agreement. Both Figures 4(a) and 4(b) demonstrate a strong agreement within limits of agreement due to 95% of the data points lying within the tight range of ±1.96 standard deviation. The path length measurements may overestimate/underestimate a surgeon's OSACSS score by 14.55 units, whilst for the log of number of movements, an overestimate/underestimate of a surgeon's OSACSS score by 68% was found. The Bland-Altman plot for number of movements to OSACSS score displayed signs of the differences being proportional to the mean, as such we take the log of number of movements and OSACSS score [15].

**Figure 5:**
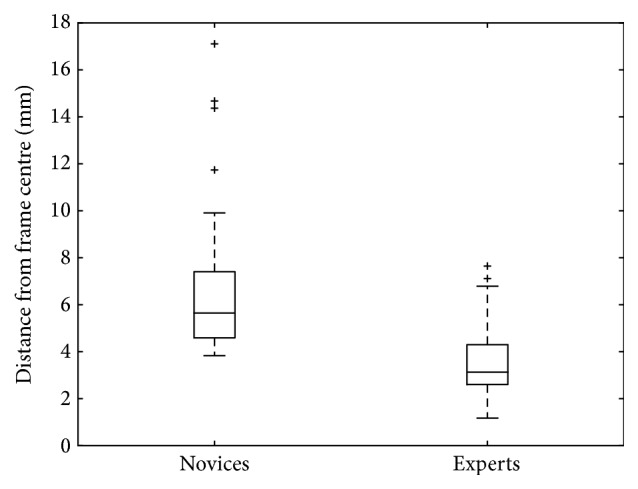
Box and whisker plot showing average pupil centre distance from frame centre between novices and experts. The horizontal line within each box is the median value, and the top and bottom borders of the box are ±1 SD with limit lines showing 95% CIs (±2 SDs).

*Group 2.* In the second group of systems, looking into operative microscope control between junior and senior surgeons, 62 participants were enrolled (31 experts, 31 novices). The comparison between novices and experts for average pupil centre to frame centre distance is shown in Figure 5. Novice surgeons had an average of 6.9 mm pupil centration with a standard deviation of 3.3 mm. In contrast, experts had good camera control minimising movements by keeping pupil centration on average 3.6 mm from the frame with a standard deviation of 1.6 mm. When analysing path length of the operative camera, novice surgeons had an average of pupil path length of 4049 mm with a standard deviation of 2709 mm. Expert surgeons had an average path length of 2512 mm with a standard deviation of 1031 mm. Hence, the novice group showed a greater total path length and a larger variation in length compared with experts who were more consistent. The *p* values for a Mann–Whitney *U* test between the two groups (novices and experts) were statistically significant at *p* ≪ 0.05.

## 4. Discussion

This study is the first in which an explicit investigation comparing two different methodologies, one human and the other machine, for measuring cataract surgical skill has been undertaken. Our results suggest a moderate correlation between surgical skills marked by a human expert as compared to the motion metrics found with computer vision algorithms. This is also the first study in which control of the operating microscope has been objectively analysed using motion analysis. A positive relationship between good camera control and surgical skill is reported.

The OSACSS was used as opposed to the ICO-OSCAR, as the original OSACSS study [1] included construct validation (the differentiation based on experience), which is directly related to the objectives of this study for comparing OSACSS with PhacoTracking and the OSACSS was designed as a quantitative rating tool. Conversely, ICO-OSCAR was designed as a formative feedback tool, designed to provide feedback and progress whilst learning and hence was less appropriate for this study.

The results presented indicate a strong link between the machine based instrument motion and the human based surgical skill rating. A higher OSACSS score was inversely proportional to the path length of the instruments [−0.68 (*p* = 0.001)], inversely proportional to the number of movements the instruments made [−0.67 (*p* = 0.002)], and inversely proportional to the total time to complete the procedure [−0.77 (*p* = 0001)]. This suggests that those with greater experience as defined by their OSACSS score [1] were more efficient in their use of instruments and quicker to complete the procedure [5]. These results are strongly in keeping with previous work in this field validating these tools. This relationship was also clearly illustrated on an individual basis, for example, in subject 16 who obtained an OSACSS score of 88, and demonstrated efficient motion (path length = 3677.771 mm, number of movements = 4646). In contrast subject 2 had a lower OSACSS score of 35 and was found to be less efficient with PhacoTrack (path length = 10146.134 mm, number of movements = 9819). Critically this is the first time that these two complementary rubrics have been evaluated on the same dataset. This may offer respective individuals a more significant amount of data by way of feedback for learning purposes than has traditionally been available to date.

The OSACCS tool is a validated method of assessing trainees' surgical competency and the level of agreement with the PhacoTrack scoring tool is illustrated in Figure 4 on the Bland-Altman plot showing agreement between the two methods, for both path lengths.  Figure 4(a) demonstrates a 14.55-point over/underestimation for path length instrument; this would not move an average surgeon's score for each cohort into a different group. In Figure 4(b) we observe that less skilled surgeons have larger percentage difference between the log total OSACSS and log of number of movements score whereas PhacoTrack overestimates the score of a novice. We hypothesise that in the case of novice surgeon's OSACSS scores are being influenced by information that does not influence the number of movements. The number of movements has an overestimation/underestimation of 68%; this would be likely to change the interpretation of a score. Hence, we strongly advocate using both human marked and motion based metrics systems concurrently, as they offer complimentary information.

The disagreements between motion based metrics and human marked metrics are not surprising. A human grader offers lower scores with the OSACCS tool when a combination of errors and inefficiencies is observed. PhacoTrack's quantitative measurements reproducibly and objectively track instruments without a surgical trainer's context. Thus, a trainee may proceed more cautiously in the context of a potentially complicated situation (and be scored appropriately and highly with OSACSS) whereas another may slow down due to inefficiency (and would score lower with OSACSS). With the purely objective PhacoTrack they may both take the same amount of time and similar number of movements and thus be rated comparably. In this paper we investigate PhacoTrack's validity in regard to its relationship to other variables and internal structure as evidence of validity provided by the original model introduced by Messick. Previous tracking work has not been undertaken in conjunction with human tools and so correlation has not been possible [9, 10, 13, 14]. Better understanding the difference in approach will potentially provide a basis to form combined measurements of surgical skill and may well prove useful in enhancing formative feedback through the two methods.

Of the 62 participants evaluated we found that novice surgeons had a higher average pupil path length and pupil centre from frame centre distance than expert surgeons who kept the surgical field through the microscope statistically significantly better centre. The measurements recorded were able to discriminate between different skill levels of surgeons and hence demonstrate construct validity (content validity is the ability of a tool to measure what it is supposed to not discriminate, not sure where this change came from). This is further exemplified by the wider spread of results found in novice surgeons. The results show that surrogate markers for camera control including lower pupil centre to frame centre measurements and a small path length all contribute to higher surgical performance. Hence, objective automated markers of both PhacoTrack parameters of instrument path length and movements and camera control data can be a useful tool for formative feedback.

Despite its crucial role in ophthalmic surgery, structured instruction of microscope camera control is often an underrepresented part of surgical training. Motion analysis of camera control may offer complementary information to be used alongside existing tools, to provide more comprehensive and structured formative feedback of microscope control intraoperatively. This technique offers an accessible system based on standard operative microscope recording equipment and, similar to simulators, an objective and numerical benchmark with an individual score breakdown (detailing parameters explaining where improvements can be made).

There are a few limitations to the study that need considering. Whilst the automated system measures efficiency of a surgeon's performance and agrees with human assessment tools, it does not provide feedback for specific tasks, which need further investigation. In addition, the automatic method does not detect any surgical errors that would be readily identified by the human observer. Whilst objective raw values of path length and number of movements provide a gross indication of surgical performance, these data values fail to identify the complexity of the case in question, the technique employed, or the critical intraoperative decision-making. We also do not know how useful these metrics are for surgeons and whether this type of feedback would improve surgical performance in the long term. This provides a further case for computational methods being used in conjunction with human based tools but further investigation will better help elucidate this.

This is the first time that an objective quantitative system has been compared with a human marked scheme for surgical skill in actual cataract surgery with promising results. Both the instrument and camera motion analysis have the potential to measure surgical skill, in a manner that is in agreement with established human methodologies. In time, automatically measured motion metrics may prove to be a useful adjunct tool to augment human marked schemes that are currently in use. The addition of a new surgical skill measure that is derived independently of existing methods can provide objective quantitative feedback to aid in the training of surgeons that complements feedback from an experienced surgeon. This is already taking place in simulators, such as the EyeSi (VR Magic, Maheim), which uses tracking metrics inside the simulated eye to form its numerical score and feedback.

Although encouraging, further work needs to be undertaken to evaluate its usefulness and applicability in surgical education and training. As motion technology develops, the operating microscope of the future may well incorporate similar algorithms allowing continuous and instant formative feedback to be available.
